# Effectiveness of the multi-component intervention ‘Focus’ on reducing smoking among students in the vocational education setting: a cluster randomized controlled trial

**DOI:** 10.1186/s12889-023-15331-5

**Published:** 2023-03-02

**Authors:** Simone G. Kjeld, Lau C. Thygesen, Dina Danielsen, Gitte S. Jakobsen, Marie P. Jensen, Teresa Holmberg, Lotus S. Bast, Lisbeth Lund, Charlotta Pisinger, Susan Andersen

**Affiliations:** 1 National Institute of Public Health, University of Southern Denmark, Studiestraede 6, 1455 Copenhagen, Denmark; 2 Center for Clinical Research and Prevention, Bispebjerg and Frederiksberg Hospital, The Capital Region of Denmark, Copenhagen, Denmark; 3 Faculty of Health Sciences, Department of Public Health, University of Copenhagen, Copenhagen, Denmark; 4 Danish Heart Foundation, Copenhagen, Denmark

**Keywords:** Smoking, Cigarettes, Smoking prevention, Intervention, Vocational schools, Cluster randomized controlled trial, Socioeconomic

## Abstract

**Background:**

Social inequality in smoking remains an important public health issue. Upper secondary schools offering vocational education and training (VET) comprise more students from lower socioeconomic backgrounds and have higher smoking prevalence than general high schools. This study examined the effects of a school-based multi-component intervention on students’ smoking.

**Methods:**

A cluster randomized controlled trial. Eligible participants were schools offering VET basic courses or preparatory basic education in Denmark, and their students. Schools were stratified by subject area and eight schools were randomly allocated to intervention (1,160 invited students; 844 analyzed) and six schools to control (1,093 invited students; 815 analyzed). The intervention program comprised smoke-free school hours, class-based activities, and access to smoking cessation support. The control group was encouraged to continue with normal practice. Primary outcomes were daily cigarette consumption and daily smoking status at student level. Secondary outcomes were determinants expected to impact smoking behavior. Outcomes were assessed in students at five-month follow-up. Analyses were by intention-to-treat and per protocol (i.e., whether the intervention was delivered as intended), adjusted for covariates measured at baseline. Moreover, subgroup analyses defined by school type, gender, age, and smoking status at baseline were performed. Multilevel regression models were used to account for the cluster design. Missing data were imputed using multiple imputations. Participants and the research team were not blinded to allocation.

**Results:**

Intention-to-treat analyses showed no intervention effect on daily cigarette consumption and daily smoking. Pre-planned subgroup analyses showed statistically significant reduction in daily smoking among girls compared with their counterparts in the control group (OR = 0.39, 95% CI: 0.16, 0.98). Per-protocol analysis suggested that schools with full intervention had higher benefits compared with the control group (daily smoking: OR = 0.44, 95% CI: 0.19, 1.02), while no marked differences were seen among schools with partial intervention.

**Conclusion:**

This study was among the first to test whether a complex, multicomponent intervention could reduce smoking in schools with high smoking risk. Results showed no overall effects. There is a great need to develop programs for this target group and it is important that they are fully implemented if an effect is to be achieved.

**Trial registration:**

https://www.isrctn.com/ISRCTN16455577, date of registration 14/06/2018.

**Supplementary Information:**

The online version contains supplementary material available at 10.1186/s12889-023-15331-5.

## Background

Despite well-documented detrimental health hazards of cigarette smoking, along with decades of efforts to reduce the prevalence and uptake of smoking globally, smoking continues to be an important public health issue [1]. While smoking rates have declined over the last decades [2, 3], the social disparities in smoking have remained rather stable. Some research even suggests increasing socioeconomic differences in smoking [4–6], i.e., individuals with lower socioeconomic status (SES) are more susceptible to initiate and maintain smoking habits [7, 8]. Consequently, focusing on individuals with lower SES is especially relevant in smoking reducing and preventive initiatives.

At the upper-secondary school level, some school types comprise a high proportion of students with low SES. In Denmark, young people who have finished compulsory education can choose between high school or vocational education and training (VET). Meanwhile, preparatory basic education (PBE) is offered to youth below 25 years who have completed compulsory school and need professional or personal upgrading to enter a youth education (e.g., VET) [9]. PBE has a strong focus on preparation for vocational education [10], and many students attending PBE choose a VET education afterward. Both VET and PBE schools have a higher uptake of low SES students compared with high schools [11], which is also reflected in smoking prevalences. Hence, in 2020, approximately 20% of students at VET and PBE schools smoked cigarettes daily, while at high schools, the corresponding prevalence was 5% [12]. These findings align with those from other Western countries [13]. Despite the pronounced differences in smoking prevalence, the proportion of youth concerned with the health risks of smoking at VET and PBE schools was on par with those attending high schools (63.0% vs. 60.6%) [12].

Within the last decades, a host of smoking preventive initiatives have been initiated on a global scale, both on a political and community-based level, as a response to the alarming trends in smoking among the general population and especially among youth [14, 15]. In particular, the school setting has been popular [15], as the opportunity of reaching youths with diverse sociodemographic characteristics is high [16]. Moreover, the school setting constitutes an important arena for engagement with peers and, hence, the social influences on smoking habits. Research suggests that possible explanations for higher smoking prevalence among VET students include higher rates of parental and peer smoking as well as the settings in which they spend their everyday lives where, i.e., smoking in school is considered a significant driver for creating social interactions among students [13, 17–19]. The evidence concerning the effect of smoking preventive and reducing interventions is inconclusive. However, interventions comprising multiple components aiming at the structural level as well as students’ competencies and knowledge about smoking have shown promising results in early and middle adolescence [20–22]. An evaluation of the settings-based intervention ‘Shaping the Social’ in Danish VET schools found that fewer students progressed from occasional to daily smoking; however, the evaluation found no intervention effects on daily smoking and smoking cessation [23]. The evaluation concluded that future preventive initiatives that also included individualized efforts were necessary when addressing this group of youth. Furthermore, another study found that current smoking was lower for students attending VET schools with smoke-free school grounds, while students attending schools allowing teacher-student smoking were more likely to smoke [24]. Thus, these findings indicate that multi-level and multi-component interventions may impact VET students’ smoking behaviors. Still, the current research on interventions to prevent and reduce smoking among older adolescents and young adults in VET settings is limited.

The primary aim of this study was to examine the effect of a school-based multi-component smoking intervention ‘Focus’ on average daily cigarette consumption among students in VET and PBE schools using a cluster randomized controlled trial. As predefined in the trial registration, we hypothesized that students would reduce the average daily number of cigarettes by 1) reduced levels of cigarette consumption among daily smokers and 2) reduced initiation of smoking and progression to daily smoking among non-smokers and occasional smokers. A secondary aim was to examine the intervention effects on a range of pre-defined secondary outcomes at the individual, interpersonal, and environmental level, e.g., individual beliefs about smoking, support from other students and class community, and visibility of smoking at school, all of which were determinants expected to impact students’ smoking decisions, identified through the program theory [25].

## Methods

### Study design

The effects of the Focus intervention were evaluated using an unblinded, cluster randomized controlled trial with stratification by school type. The study is prospectively registered as ISRCTN16455577 in the ISRCTN trial registry. This study is reported according to the Consolidated Standards of Reporting Trials (CONSORT) extension to cluster randomized controlled trials [26] and Template for Intervention Description and Replication (TIDieR) recommendations [27]. A detailed study protocol is published elsewhere [25].

### The Focus intervention

The Focus intervention is a multi-component school-based intervention aiming to reduce smoking among students at VET and PBE schools. The intervention was developed based on the Intervention Mapping framework [28] and the Behavior Change Wheel model [29] guided by self-determination theory [30] and socio-ecological theories [31, 32]. The intervention included the following six main components:

1. *School smoking policy*. Schools were encouraged to implement a strict school smoking policy, i.e., enforcement of smoke-free school hours, where neither staff nor students are allowed to smoke during school hours. Smoke-free school hours included completely smoke-free indoor school facilities and outdoors during school hours (a fixed time interval decided by the school management, e.g., from 8 a.m. to 4 p.m.) effective for all students, staff, and visitors at the school.
2. *A two-day course for school staff in delivering short motivational counseling with young individuals about smoking*. This course was offered to 2–4 teachers or other relevant staff at the intervention schools and was arranged by the Danish Cancer Society in the planning phase.
3. *School class-based teaching material*. The teaching material consisted of eight sessions about attitudes, beliefs, and social influence regarding smoking and aimed to create reflection in students which may lead to awareness and motivation to reduce, quit, or not initiate smoking.
4. *Edutainment session on smoking*. Edutainment refers to a lecture that both educates and entertains as defined by the American Heritage Dictionary [33]. The edutainment session was a one-time event for students performed by an external professional actor to promote knowledge about nicotine dependency, the consequences of smoking, individual risk of illness, and common misperceptions about smoking delivered with a combination of information and entertainment.
5. *Class-based competition based on measurements of carbon monoxide levels*. The competition included measurements of carbon monoxide levels at school start (baseline) and after two months. Here, researchers visited schools during school hours and carried out the measurements among students. Students were to breathe into a carbon monoxide detector, in which their level of carbon monoxide was measured and noted. The class with the largest overall reduction or maintenance of carbon monoxide level during the period won a prize (a bowling tour for the class).
6. *Access to smoking cessation support*. Individual smoking cessation support was offered by a smoking cessation counselor from the National Quitline in Denmark (www.stoplinien.dk) introduced to the students in the edutainment session.

The intervention targeted the youngest students at the schools, i.e., basic course students at VET schools and all students at PBE schools. Schools in the control group were encouraged to continue with normal practice, e.g., class-based educational material on smoking, but no school-level smoke policies in the form of smoke-free school hours.

## Setting and participants

The VET-system comprises a basic program, in two parts, followed by a main program. Each part of the basic program has a duration of 20 weeks (i.e., one semester), with the first part of the basic course aimed at students who left compulsory school since maximum two years, while the second part includes everyone and is an introduction to the main program. VET is divided into four main areas: (1) social and healthcare, (2) commercial, (3) agriculture, and (4) technical [10]. We did not include the third arm because these schools have the educational facility on a working college farm.

Schools were recruited in two rounds, from January 2018 to April 2018 and January 2019 to April 2019. These time frames were selected to ensure that schools had time to implement the Focus intervention at school start in August. In the first round, seven VET schools were enrolled and randomly assigned to the intervention group (n = 4) or control group (n = 3). It was difficult to recruit VET schools with ‘technology, construction, and transportation’ courses. Therefore, in round two, PBE schools were also included. A total of three VET schools and four PBE schools were enrolled and randomly assigned to the intervention group (n = 4; two PBE schools and two VET schools) or control group (n = 3; two PBE schools and one VET school). One PBE school withdrew their participation and was replaced.

The five inclusion criteria were (a) VET school offering education within social and healthcare, commercial, or technical subject areas or PBE school, (b) at least 100 students enrolled in the basic program (first school year) at VET schools or in total at PBE schools, (c) no school-level smoke policies in the form of smoke-free school hours, (d) school management was willing to implement the Focus intervention, and (e) accept the randomization procedures of the intervention. The VET schools were randomly selected by identifying schools fulfilling the inclusion criteria based on data from the student registry whereafter we placed them in random order regarding the sequence of contacting the schools. Due to time constraints, the PBE schools were selected based upon convenience sampling. This involved that we contacted the Danish association for PBE schools as well as a manager of a PBE school who had already implemented smoke-free school hours. We asked these central stakeholders to help point in the direction of schools who could be interested in participating in the intervention.

At the VET intervention schools, data collection was performed in the basic course classes where the class-based intervention components were implemented. At the VET control schools, data collection was also performed in the basic course classes. Data collection was performed among all students in PBE schools as the intervention was implemented in all classes.

### Randomization

We randomized at the school level to avoid contamination in the control group. The randomization, stratified by school type, was used to assign schools to the intervention or the control group. At each round, VET schools were divided into the following strata: (1) social and healthcare, (2) commercial, and (3) technical. In the second round, a fourth stratum with PBE schools was added. A statistician not involved in the study performed randomization within each stratum and informed the project leader (SA). The project team informed schools by e-mail and visited the intervention schools to plan and support implementation.

### Measures

Baseline data were collected at the semester start after the summer holiday (August). Here, students completed a digital questionnaire during school hours about smoking, attitudes towards smoking, sociodemographic questions as well as other health and well-being related questions. At the same time, teachers and principals answered a baseline data questionnaire about smoking at their schools, rules, etc. A longitudinal follow-up assessment was performed approximately five months after baseline among students (December), and prior to this follow-up assessment, school principals and teachers in intervention classes answered a questionnaire about the implementation of the intervention (November).

All question framings and definitions of variables used in this study are in detail described in Additional file 1.

#### Primary outcomes

In the prospective trial registration, the primary outcome measure was stated as the ‘number of cigarettes per day’, assessed by student questionnaire at baseline and post-intervention. For that purpose, we needed a model that included the whole student population to understand the intervention effect on daily cigarette consumption in full. We could have used a zero-inflated model, as these are designed to deal with data with a large number of individuals with a count of zero (i.e., students reporting not to smoke cigarettes on a daily basis). Because of fit and interpretation difficulties by using a zero-inflated model, we decided that two separate models were more appropriate. To accommodate students who reported zero number of daily cigarettes at follow-up, but reported daily smoking at baseline, we decided to have an additional primary smoking outcome. This was, however, not the original primary outcome choice. Following this, two primary outcomes were assessed in this paper:

1. *Daily cigarette consumption* among daily smokers at follow-up. Students who reported to smoke daily were given the question ‘how many cigarettes do you typically smoke per day? (State the average number of cigarettes)’.
2. *Daily smoking* at follow-up. All students were given the question ‘do you smoke cigarettes’ with response options of daily, weekly, less than weekly, or not smoking currently, which were dichotomized into daily smoking vs. non-daily smoking.

We defined a supplementary *smoking status* outcome regarding *regular smoking.* Responses to ‘do you smoke cigarettes?’ was dichotomized into regular smoking (daily, weekly, less than weekly) vs. non-smoking (non-smoking or previous smoking), respectively.

#### Secondary outcomes

The secondary outcomes were determinants expected to impact students’ smoking behaviors as prespecified in the trial registration and study protocol [25]. Some outcomes were examined among all students, while others were restricted to groups with specific smoking status at follow-up.

Among daily smokers, cigarettes smoked during school hours and nicotine dependence were measured at follow-up. Among regular smokers, outcomes such as intention to quit smoking, self-efficacy, smoking behavior at school, and knowledge about smoking cessation services were measured at follow-up. Among non-smokers, intention to initiate smoking was examined at follow-up. Finally, among all students, outcomes at follow-up included mood effects of smoking, social benefit effects of smoking, school well-being, and visibility and perception of smoking at school and among classmates.

#### Covariates

Baseline characteristics included gender, age, and socio-economic position (SEP). SEP was assessed according to family occupational social class determined by two questions about the occupations of students’ father and mother and categorized into low, medium, and high SEP. Previous research has demonstrated that students can answer questions about parental occupational status with fair validity [34].

#### Implementation measure

A web-based survey was delivered to school principals about the implementation of the intervention components approximately four months (November) after baseline (August). Based on these responses, a variable was derived about the implementation level. Categorizations of schools were divided into (1) implementation of both school- and class-level intervention components (intervention delivered in full), (2) or only some class-level components or withdrawal of components during the evaluation period (partial intervention), and (3) control schools.

### Sample size

Sample size calculations indicated that a sample size of 10 schools with an average of 100 students (e.g., five schools in the intervention group and five schools in the control group) was necessary to detect a 25% reduction in the number of cigarettes smoked daily at follow-up, with a power of 80% and an alpha of 5%. The cluster design was taken into account with an assumed intra-cluster correlation coefficient for schools (ICC) of 0.025. The ICC was estimated based on the Danish National Youth Study 2014 data [35]. With the assumption of 25% student non-response, 1,250 total students were needed. Considering the risk of attrition at school level, we enrolled more schools (n = 14).

### Statistical methods

Statistical analyses were conducted using SAS v. 9.4. Descriptive statistics were used to compare intervention and control groups at baseline. An intention-to-treat approach was used in the analyses of all trial outcomes, and all students with baseline data information at all participating schools were included in the analyses. Students who reported smoking above 65 cigarettes per day at baseline and follow-up and concurrently answered the highest response options to other questions (e.g., reported believing that all students at their schools smoked cigarettes, smoked the maximum number of cigarettes during school days, i.e., 30 cigarettes, or gave unserious answers in open-text questions), thus, indicating sabotage of survey responses, were excluded for further analyses (n = 4).

Missing data were handled with multiple imputation in two steps. First, the missing values in the baseline data were imputed. Next, missing follow-up data were imputed based on baseline data and non-missing values for primary and secondary outcomes at follow-up. Using the PROC MI procedure in SAS, two times of 20 rounds of imputations were carried out. In the first round, the school class variable was not included in the imputation model, while in the second round, the school class variable was included in the imputation model to account for dependency within school classes. Using SAS PROC MIANALYZE, the imputed datasets were summarized to estimate intervention effects. Complete case analysis was utilized as a sensitivity analysis; the results did not differ substantially and therefore, only the intention-to-treat results are presented.

The effect analyses were based on between-group comparisons based on the five-month test and adjusted by the baseline score of the outcome variable if measured, and baseline age, gender, and SEP as covariates to increase the precision of effect estimates. The stratification variable, school type, was also included as a covariate. Each model included class as a random effect to account for the cluster effect. Class-level intraclass correlations (ICC) are presented for the smoking outcomes. Multilevel general linear regression modeling estimated the association between the intervention and daily cigarette consumption among daily smokers. We graphically checked model assumptions of normal distribution and variance homogeneity of the residuals that indicated influential outliers that violated the assumptions. We examined these outliers, and the values were all above 25 cigarettes per day (range: 28–48 cigarettes). Therefore, we performed a sensitivity analysis in which we excluded students with values above 25 (n = 8), resulting in a reasonable model fit (see results in Additional file 2). The dichotomous outcomes, i.e., smoking status and secondary outcomes, were assessed with multilevel logistic regression models.

We examined preplanned subgroups within intervention schools (baseline smoking status, age, gender, and school type) and possible moderating effects on the daily cigarette consumption and smoking status at follow-up. Baseline smoking status was categorized into daily smoking, regular smoking, and non-smoking. Age was divided into < 18 years and ≥ 18 years. School type was categorized as (1) VET schools: social and healthcare, (2) VET schools: technical and commercial, and (3) PBE schools. Moreover, we conducted a per-protocol analysis to investigate the effect of the actually delivered intervention.

## Results

### Baseline characteristics

**Fig. 1:**
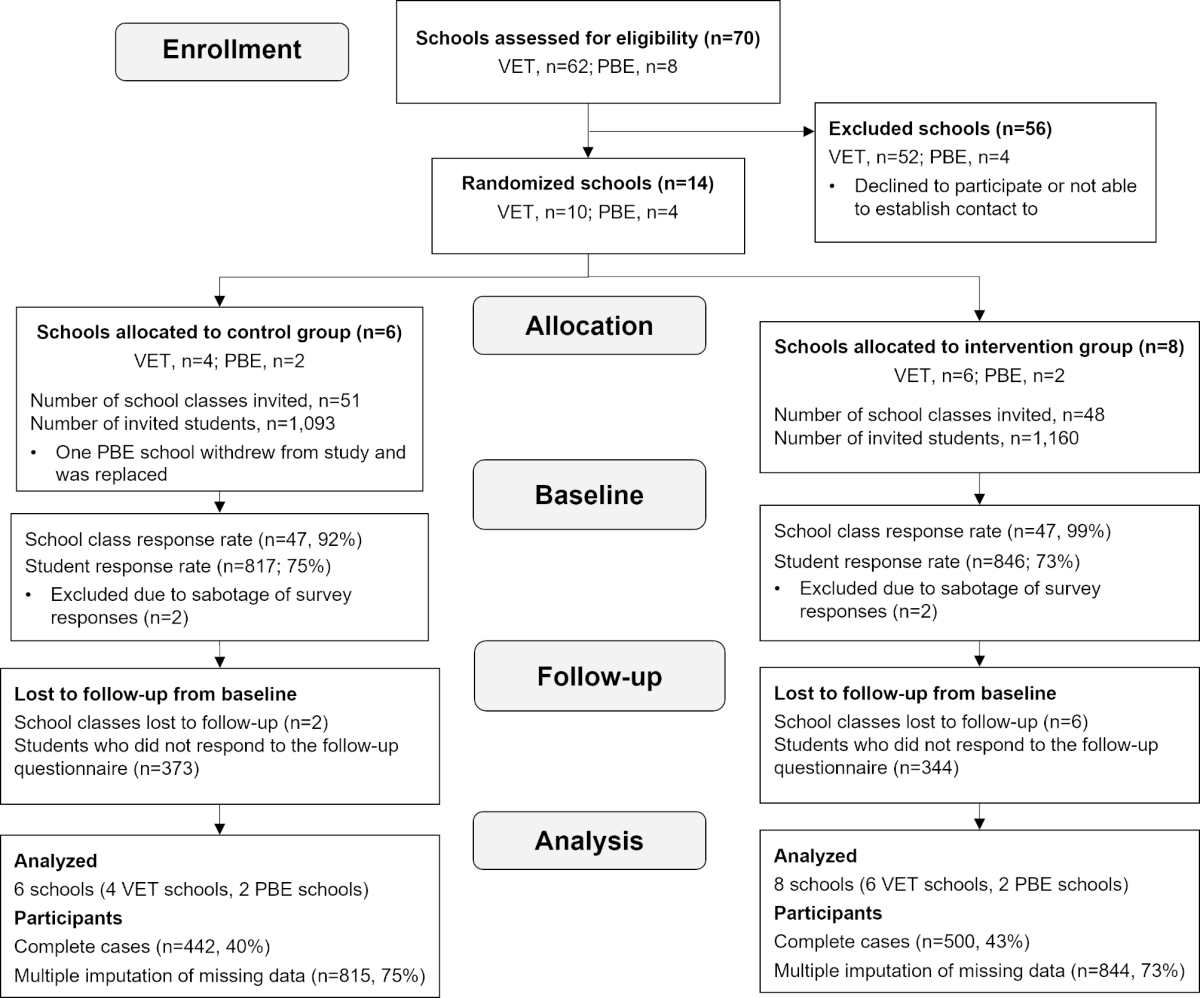
Flow diagram

At baseline, the survey sample included 844 students in the intervention group (response proportion 76%) and 815 in the control group (response proportion 75%). See Fig. 1 for a full overview of the participant flow.

Baseline characteristics of the students are summarized in Table 1. The students were on average 18 years old, and 30% smoked daily, while 11% smoked occasionally.

**Table 1:** Baseline characteristics of schools and students stratified by the intervention and control group, complete cases, N = 1,659

	Total	Control group	Intervention group
	n (%)	n (%)	n (%)
School characteristics			
Number of schools, n	14	6	8
School type			
VET schools: social and healthcare	5 (35.7)	2 (33.3)	3 (37.5)
VET schools: technical	3 (21.4)	1 (16.7)	2 (25.0)
VET schools: commercial	2 (14.3)	1 (16.7)	1 (12.5)
PBE schools	4 (28.6)	2 (33.3)	2 (25.0)
Average number of classes per school [mean, (SD)]	6.6 (3.6)	5.9 (2.0)	7.5 (4.9)
Average number of students per class [mean, (SD)]	16.3 (10.1)	17.4 (8.4)	15.6 (11.1)
**Student characteristics**			
Number of students, n	1,659	815	844
Age, years [median (IQR)]	18.0 (17;22)	17.0 (17;20)	18.0 (17;22)
Women	833 (50.2)	390 (47.9)	443 (52.5)
Immigrant background			
Danish origin	1,272 (77.8)	650 (80.8)	622 (74.9)
Descendant	97 (5.9)	45 (5.6)	52 (6.3)
Immigrant	266 (16.3)	110 (13.7)	156 (18.8)
Living arrangement			
Living with both parents	629 (38.3)	301 (37.3)	328 (39.2)
Living with one parent (parents not living together)	464 (28.2)	258 (32.0)	206 (24.6)
Other (alone, with partner, with friends etc.)	550 (33.5)	247 (30.7)	303 (36.2)
Family occupational social class			
High (I + II)	251 (15.3)	120 (14.9)	131 (15.6)
Middle (III + IV)	678 (41.2)	355 (43.9)	323 (38.5)
Low (V + VI)	423 (25.7)	201 (24.9)	222 (26.5)
Unclassifiable	251 (15.3)	132 (16.3)	162 (19.3)
Baseline smoking status			
Daily smoking	491 (29.7)	227 (27.9)	264 (31.3)
Occasional smoking	181 (10.9)	92 (11.3)	89 (10.6)
Previous smoking	102 (6.2)	56 (6.9)	46 (5.5)
Tried once or twice	263 (15.9)	136 (16.7)	127 (15.1)
Never smoked	619 (37.4)	302 (37.2)	317 (37.6)
Daily cigarette consumption [mean, (SD)]^a^	12.8 (7.06)	12.7 (6.1)	13.9 (6.9)
Medium to high nicotine dependence^a^	152 (63.3)	63 (59.4)	89 (66.4)
Smoking prevalence among family and friends			
One or both parents smoke (incl. stepparents)	841 (50.7)	405 (49.7)	436 (51.7)
One or more siblings smoke	546 (32.9)	274 (33.6)	272 (32.2)
At least one of my close friends/partner smokes	1,241 (74.8)	609 (74.7)	632 (74.9)

*Note*. Baseline characteristics among complete cases with a missing range on included variables between 0.2-1.5%

^a^among students who smoke daily

### Intervention effects on number of cigarettes and smoking status

Among daily smokers, daily cigarette consumption decreased slightly between baseline and follow-up in the control group (from 12.7 to 12.2) and the intervention group (14.1 to 13.7) with a statistically non-significant mean difference of 0.15 (95% CI: -1.25;1.54) between the intervention and control group (Table 2). Among daily and regular smokers, no statistically significant between-group differences were found at follow-up. However, diverging tendencies were found in daily smoking with a slight reduction in the intervention group (from 31.3 to 30.1%) and a slight increase in the control group (from 27.7 to 28.5%). The adjusted analysis showed a OR of 0.59 (95% CI: 0.30;1.15) in favor of the intervention.

**Table 2:** Analyses of the associations between the intervention (versus control group) and daily smoking consumption as well as smoking status (daily and regular) at follow-up using multiple imputation of missing data (N = 1,659)

Outcomes	Baseline	Follow-up	Baseline adjusted*			Fully adjusted**			Class-level ICC	
Daily cigarette consumption (40xN = 475–495)^a^	Mean (SD)	Mean (SD)	Mean difference^b^	CI (95%)	p-value	Mean difference^b^	CI (95%)	p-value		
Control group	12.7 (6.1)	12.2 (6.9)	0	(ref)		0	(ref)		3.4%	
Intervention group	14.1 (7.0)	13.7 (8.3)	0.12	-1.25;1.48	0.87	0.15	-1.25;1.54	0.84		
**Daily smoking (40xN = 1,659)**	**%**	**%**	**OR**	**CI (95%)**	**p-value**	**OR**	**CI (95%)**	**p-value**		
Control group	27.7	28.5	1.00	(ref)		1.00	(ref)		4.5%	
Intervention group	31.3	30.1	0.60	0.31;1.16	0.13	0.59	0.30;1.15	0.12		
**Regular smoking** **(40xN = 1,659)**	**%**	**%**	**OR**	**CI (95%)**	**p-value**	**OR**	**CI (95%)**	**p-value**		
Control group	39.0	39.4	1.00	(ref)		1.00	(ref)		1.8%	
Intervention group	41.9	40.0	0.76	0.48;1.19	0.23	0.77	0.49;1.20	0.24		

*adjusted for random effects (class), stratification variable (school type), baseline smoking status and cigarette consumption

**adjusted for random effects (class), stratification variable (school type), baseline smoking status, cigarette consumption, gender, socioeconomic status, and age

^a^number differed due to the multiple imputation models

^b^mean difference in change from baseline to follow-up

### Per-protocol analysis

The per-protocol analysis showed no statistically significant differences in the average number of cigarettes smoked, daily smoking, nor regular smoking between the intervention and control group (Table 3). However, a consistent tendency was found for all outcomes where the intervention was delivered in full, i.e., a reduction of average daily cigarette consumption (mean difference: -0.66, 95% CI: -2.42;1.09), daily smoking (OR: 0.44; 95% CI: 0.19;1.02), and regular smoking (OR: 0.57; 95% CI: 0.32;1.01). while no marked differences were observed between schools where the intervention was delivered partially and the control group.

**Table 3:** Per-protocol analysis using multiple imputation of missing data (N = 1,659)

	Baseline	Follow-up	Fully adjusted*	
Daily cigarette consumption	Mean (SD)	Mean (SD)	Mean difference^a^	CI (95%)
Per protocol analysis				
Control group	12.7 (6.1)	12.2 (6.9)	0	(ref)
Partial intervention	14.7 (7.5)	14.6 (9.1)	0.88	-0.77;2.53
Full intervention	13.4 (6.4)	12.8 (7.3)	-0.66	-2.42;1.09
**Daily smoking**	**%**	**%**	**OR**	**CI (95%)**
Per protocol analysis				
Control group	27.7	28.6	1.00	(ref)
Partial intervention	31.4	31.1	0.78	0.35;1.76
Full intervention	31.2	29.2	0.44	0.19;1.02
**Regular smoking**	**%**	**%**	**OR**	**CI (95%)**
Per protocol analysis				
Control group	39.0	39.4	1.00	(ref)
Partial intervention	42.5	42.6	1.00	0.58;1.72
Full intervention	41.3	37.4	0.57	0.32;1.01

*adjusted for random effects (class), stratification variable (school type), baseline smoking status, cigarette consumption, gender, socioeconomic status, and age

^**a**^mean difference in change from baseline to follow-up

### Differential effects by gender, age, baseline smoking status, and school type

For all subgroup analyses, no statistically significant differences were found between the intervention and control groups in relation to daily cigarette consumption (Table 4). Girls in the intervention group were significantly less likely to smoke daily at follow-up (OR: 0.39; 95% CI: 0.16;0.98) and regularly (OR: 0.37; 95% CI: 0.18;0.79) compared with girls in the control group (Table 5). Regarding school type, a statistically significant reduction in regular smoking (OR: 0.38; 95% CI: 0.14;0.98) among students at social and healthcare VET schools was found in the intervention group compared with students at social and healthcare VET control schools.

**Table 4:** Subgroup analyses stratified by gender, age, and smoking status at baseline using multiple imputation of missing data (N = 1,659)

	Control group		Intervention group		Regression analyses	
	Baseline	Follow-up	Baseline	Follow-up	Fully adjusted*	
Daily cigarette consumption	Mean (SD)	Mean (SD)	Mean (SD)	Mean (SD)	Mean difference^a^	CI (95%)
Gender						
Boys	12.8 (6.3)	12.6 (7.1)	14.2 (7.4)	14.2 (9.0)	0.81	-1.35;2.96
Girls	12.6 (5.9)	11.9 (6.7)	14.0 (6.7)	13.3 (7.6)	-0.68	-2.03;0.67
Age, years						
< 18	12.5 (5.6)	11.7 (7.1)	13.1 (6.7)	13.5 (7.6)	0.65	-1.65;2.94
≥ 18	12.8 (6.3)	12.5 (6.6)	14.6 (7.2)	13.9 (8.7)	-0.33	-1.77;1.11
Smoking status at baseline						
Daily smoking	12.7 (6.1)	12.5 (6.8)	14.1 (7.0)	13.9 (8.4)	1.14	-0.69;2.98
Occasional smoking	-	10.2 (8.1)	-	9.8 (6.2)	-2.33	-11.25;6.59
No current smoking	-	6.6 (3.6)	-	14.0 (4.3)	-^b^	
School type						
VET schools: Social and healthcare	12.7 (5.2)	11.8 (5.6)	13.9 (6.6)	13.2 (7.5)	-0.34	-1.82;1.14
VET schools: Technical and commercial	11.9 (5.8)	11.4 (6.8)	12.6 (6.1)	13.0 (7.2)	0.96	-1.97;3.90
PBE schools	14.0 (7.6)	14.2 (8.4)	15.8 (8.1)	15.1 (9.9)	-0.38	-3.05;2.29
**Daily smoking**	**%**	**%**	**%**	**%**	**OR**	**CI (95%)**
Gender						
Boys	26.6	26.0	30.7	29.2	0.76	0.33;1.76
Girls	28.9	31.3	31.8	31.0	0.39	0.16;0.98
Age, years						
< 18	19.6	22.9	22.0	22.1	0.51	0.22;1.15
≥ 18	37.3	35.3	40.1	37.7	0.90	0.38;2.12
Smoking status at baseline						
Daily smoking	100	93.8	100	91.3	0.62	0.28;1.37
Occasional smoking	0	13.4	0	11.3	0.96	0.31;2.93
No current smoking	0	1.7	0	0.6	-^b^	
School type						
VET schools: Social and healthcare	29.8	31.8	36.8	34.7	0.52	0.18;1.49
VET schools: Technical and commercial	21.9	23.0	22.1	21.4	0.64	0.23;1.84
PBE schools	41.3	39.1	38.6	37.8	0.41	0.10;1.77
**Regular smoking**	**%**	**%**	**%**	**%**	**OR**	**CI (95%)**
Gender						
Boys	40.1	38.9	43.7	43.6	1.21	0.72;2.05
Girls	37.9	39.9	40.2	36.8	0.37	0.18;0.79
Age, years						
< 18	34.6	35.7	37.2	36.7	0.87	0.53;1.44
≥ 18	44.3	43.7	46.3	43.2	0.54	0.22;1.28
Smoking status at baseline						
Daily smoking	100	96.0	100	94.4	0.67	0.27;1.69
Occasional smoking	100	77.6	100	70.0	0.68	0.31;1.47
No current smoking	0	6.6	0	5.3	0.83	0.45;1.53
School type						
VET schools: Social and healthcare	38.6	40.7	45.8	40.6	0.38	0.14;0.98
VET schools: Technical and commercial	37.4	36.8	36.5	36.9	1.13	0.67;1.91
PBE schools	45.2	44.8	45.1	44.4	0.58	0.03;10.3

*adjusted for random effects (class), stratification variable (school type), baseline smoking status, cigarette consumption, gender, socioeconomic status, and age

^**a**^mean difference in change from baseline to follow-up

^b^there are too few observations in some cells to run analysis

### Intervention effects on secondary outcomes

For mood effects of smoking, students in the intervention group showed improvement compared with the control group (OR: 0.68; 95% CI: 0.47;0.99). For all other secondary outcomes, no statistically significant differences were found between the intervention and control group (Table 5).

**Table 5:** Logistic regression analyses of the associations between the intervention group (versus the control group) and secondary outcomes of the Focus intervention at follow-up using multiple imputation of missing data (N = 1,659)

	Control group		Intervention group		Regression analyses	
Outcome	Baseline	Follow-up	Baseline	Follow-up	Fully adjusted*	
Among daily smokers	Mean (SD)	Mean (SD)	Mean (SD)	Mean (SD)	Mean difference^a^	CI (95%)
Cigarettes smoked during school hours	-	5.4 (3.2)	-	6.1 (4.1)	0.23	-0.94;0.66
	**%**	**%**	**%**	**%**	**OR**	**CI (95%)**
Medium to high nicotine dependence	75.6	72.4	79.3	77.2	1.09	0.59;2.01
**Among regular smokers**	**%**	**%**	**%**	**%**	**OR**	**CI (95%)**
Intention to quit smoking	69.7	77.2	70.5	79.0	1.18	0.64;2.19
Smoking-related self-efficacy (scale)	13.1	14.1	13.8	13.7	0.95	0.65;1.39
Smoking-related self-efficacy at school	-	43.4	-	42.5	0.98	0.63;1.53
Smoking together with teachers while at school	-	10.0	-	10.1	1.15	0.51;2.55
Smoking together with other students while at school	-	51.9	-	58.5	1.38	0.85;2.25
Smoking alone while at school	-	16.0	-	18.3	1.17	0.63;2.19
Knowledge about smoking cessation services	14.3	16.3	12.7	17.2	1.10	0.60;1.98
**Among non-smokers**	**%**	**%**	**%**	**%**	**OR**	**CI (95%)**
Intention to initiate smoking	2.7	5.5	3.5	5.2	0.86	0.43;1.70
**Among all**	**%**	**%**	**%**	**%**	**OR**	**CI (95%)**
Perceived mood effects of smoking	23.4	34.4	26.3	31.2	0.68	0.47;0.99
Perceived social benefit effects of smoking (scale)	-	3.2	-	2.6	1.01	0.43;2.35
Positive classmate relationships (scale)	-	62.7	-	60.9	0.91	0.62;1.35
Sense of class community	-	52.2	-	54.4	1.08	0.71;1.64
School connectedness	-	57.0	-	57.9	1.02	0.72;1.46
Support from other students	-	65.7	-	60.5	0.79	0.53;1.19
Support from teachers	-	65.9	-	65.3	1.10	0.73;1.66
Afraid of being made a fool of	-	11.8	-	11.5	1.03	0.63;1.70
Visibility of smoking among all students at the school (≥ 50%)	-	48.9	-	47.1	0.87	0.58;1.31
Visibility of smoking among classmates (≥ 50%)	-	43.0	-	42.3	0.95	0.57;1.57
Perception of how many smokes (> 50%)	-	39.2	-	36.0	0.79	0.52;1.20

*adjusted for random effects (class), stratification variable (school type), gender, socioeconomic status, age, baseline variable if available, and baseline smoking status

## Discussion

This study examined the effectiveness of a multi-component and multi-level intervention to reduce smoking among young people in upper-secondary schools with high smoking prevalences (Danish VET and PBE schools). The results showed no effects of the intervention on the primary outcomes, i.e., daily cigarette consumption and students’ smoking status. However, a rather consistent tendency was found that students in the intervention group were less likely to smoke at follow-up compared with schools in the control group. Subgroup analyses showed that the intervention effect differed according to gender, school type, and level of implementation. Specifically, intervention effects were seen for girls as well as social and healthcare VET schools – although the size of effect was relatively minor. Moreover, schools where the intervention was delivered in full had higher benefits of the intervention in terms of reduced smoking prevalence at follow-up than schools with partial delivered intervention.

Most school-based tobacco interventions have focused on younger adolescents and the primary school setting [15]. Thus, findings from this study add to the current evidence of the potential effects of multi-component and multi-level interventions. This study indicates that smoking preventive initiatives may impact smoking decisions among adolescents and young adults at higher risk of smoking in the VET setting, which is in line with the sparse previous research in this area [23, 24]. Meanwhile, a range of factors may have influenced the opportunities to implement intervention components as intended. In a Danish and international context, various barriers have been identified for implementing school tobacco policies [36, 37]. These factors include, e.g., lack of sanctioning of smokers at school due to fear of jeopardizing the student-teacher relationship, lack of clear communication from management on enforcement of smoking rules as well as perceiving smoking as outside their role as school staff and as students’ own responsibility. Seeing smoking policies in line with school values and expecting positive outcomes from the policies were found to be important facilitators.

In a qualitative process evaluation of the Focus intervention, we saw that VET schools educating health personnel (the social and healthcare trade) were more prone to implement and make sense of the intervention [37]. The current study underpins the importance of these observations, namely that students at social and healthcare VET schools in the intervention group were less likely to smoke at follow-up compared with their counterparts in the control group, while students attending other schools (technical and commercial VET schools as well as PBE schools) did not achieve the same positive outcomes.

The intervention was more effective on girls’ smoking behavior than boys. Consequently, the intervention may have appealed more to girls than boys, which is important to bear in mind when interpreting the results. Other research has shown similar gender effects of health interventions and intervention adoption; thus, girls were more responsive to smoking preventive interventions and had more positive attitudes towards tobacco policies [38]. Future research should be aware of these differences and further address how to design intervention components to appeal to boys and girls equally. The fact that intervention effects were seen for girls, social and healthcare VET schools, and schools where the intervention was implemented in full, may reflect the same underlying finding; hence, the proportion of girls at social and healthcare VET schools is high compared to the other school types, and most of the social and healthcare VET schools had implemented the intervention in full. Due to limited power, further subgroup analyses based on gender within school types were not possible. Nonetheless, the findings speak to further actions within the VET setting and a focus on tailoring efforts to the specific VET setting so that all schools, even without a particular focus on health, can make sense of and perceive smoking preventive interventions to be in line with their school values and educational aims.

Knowledge about the effects of smoking prevention in high-risk groups are lacking, and evidence-based knowledge may inform and guide political decision-making. We know that smoking prevention as early as possible is important to prevent individuals to initiate smoking, i.e., as early as primary school. Nonetheless, many, especially in the VET setting, escalate smoking when they enter upper-secondary education [39]. Therefore, efforts within the setting of youth in high risk of smoking is essential to de-normalize smoking and provide youth with tools to abstain from smoking and social alternatives to social communities connected to smoking. Importantly, this study showed that implementation is essential for achieving positive outcomes of the intervention. Future studies may further investigate other aspects of the implementation, namely in-depth research into e.g., the enforcement of the intervention and attitudes towards components of the intervention among students and specific subgroups could be beneficial to gain an overall picture of how and for whom the intervention works. Societal norms and practices regarding smoking are essential for succeeding in implementing new smoking preventive interventions. Since the Focus study was implemented and evaluated, smoke-free school hours schools at VET schools have been made mandatory by the Danish government, and the laws on tobacco have generally been substantially tightened over the recent years [40]. Therefore, new research is warranted to further investigate youth smoking practices and the potentials of the Focus intervention in light of this new policy environment in Denmark.

### Strengths and limitations

This study holds several strengths, including that the Focus intervention was evaluated in a longitudinal cluster-randomized controlled trial with a large baseline sample of youth. Moreover, advanced statistical methods were used to account for clustering and missing data. Secondly, the development of the Focus intervention builds on a large and thorough preparation work, including a needs assessment and a clearly defined program theory of how the intervention was expected to impact students’ smoking decisions [25]. Moreover, the intervention comprised components figuring on the structural and individual level, and thereby addressing smoking determined not only by students’ individual behaviors but also by their social determinants [41], highlighting the importance of a socio-ecological approach for preventing tobacco use among young people. Thirdly, the study fills an important knowledge gap identified in the current literature on smoking prevention, as this study focuses on the effects of an approach on preventing the escalation of smoking targeting high-risk populations [42], i.e., students at VET and PBE schools.

Nonetheless, findings from this study should be considered in the context of the following limitations. First, a large proportion of students missed responding at follow-up. To account for potential selection bias, we performed analyses based on multiple imputation including a range of relevant factors. Nevertheless, missing data reduced statistical power with a risk of introducing type II error. Second, the lack of statistically significant results may be influenced by the short follow-up period in which students’ smoking behaviors were investigated. Changing habits and culture in any setting is a difficult task, and it may take a longer time to find common ground on a managerial level on how to address tobacco-related issues and develop new habits. In this connection, a systematic review among schoolchildren found an effect of smoking preventive interventions at more than one-year follow-up, while no effects were detected at one year or less [15]. Third, use of self-reported measures may have introduced information bias. Particularly, responses by school principals may have been influenced by a desire to present the school in a favorable way. Fourth, we tested intervention effects on a large number of outcomes which may have introduced Type I error. To take this into account, we had pre-specified a program theory and only included a few primary outcomes. We only performed power calculations for the cigarette consumption outcome. Consequently, it is unknown whether we have sufficient power for the analysis of the daily smoking outcome. Considering the risk of attrition at school level, we chose to enroll more schools than the power calculation estimated (n = 14). This was also done to ensure power for the subgroup analyses. However, the study was not formally powered for subgroup analyses and, therefore, the pre-planned subgroup analyses should be considered exploratory.

Finally, recruiting VET schools showed to be a difficult task, and PBE schools were included due to the many similarities among students attending VET and PBE schools – both according to sociodemographic characteristics and smoking prevalence. Nonetheless, as were also reflected in our results, students in PBE schools could constitute a more vulnerable group (i.e., smoking prevalence was markedly higher among students at PBE schools compared with students at VET schools). Therefore, components of the intervention may not have applied equally to students of different school types. Future studies may further investigate the contextual influences on the effects of smoking preventive interventions.

Because we had the inclusion criteria that the school management should be willing to implement the Focus intervention, we expect differences in the management group between included schools and schools who rejected our invitation to participate. The schools had different reasons for the rejection including that their focus and resources were not able to meet the requirement of implementing smoke-free school hours. Accordingly, the engagement in implementing the school tobacco policy cannot be generalized to all schools in Denmark. However, we do not have reasons to suspect that the student population is not representative for vocational students in Denmark, i.e., in another Danish report from 2019 among VET schools, the smoking prevalence was comparable with our study (30% smoked daily and 11% smoked occasionally in VET schools in this study vs. 29% and 9%, respectively) [43].

## Conclusion

This study showed no overall intervention effects on daily cigarette consumption and smoking status. Still, results based on subgroup analyses indicated some context-specific effects. Hence, our results showed effects in social and health care VET schools and among girls. Moreover, findings suggested that smoking can be reduced if the intervention is fully implemented with both individual and structural components at the school level. We found inconclusive evidence on the investigated secondary outcomes that were determinants expected to impact smoking decisions, and future studies should further examine the mechanisms contributing to reduced smoking among some groups of youth and why other groups maintain their smoking habits.

## Electronic supplementary material

Below is the link to the electronic supplementary material.

Supplementary Material 1

Supplementary Material 2

## Data Availability

Data that support the findings of this study are available from University of Southern Denmark (SDU). Restrictions apply to the availability of data which were used under license for the current study and are thus, not publicly available. Data are available from the corresponding author upon reasonable request and with permission from SDU.

## References

[1] US Department of Health Human Services (2014). The health consequences of smoking—50 years of progress: a report of the Surgeon General.

[2] Organization WH. WHO global report on trends in prevalence of tobacco use 2000–2025. World Health Organization; 2019.

[3] Hoffmann SH, Schramm S, Jarlstrup NS, Christensen AI, Danskernes rygevaner (2018). Udvikling fra 1994 til 2017. [Smoking habits among danish citizens. The development from 1994 to 2017].

[4] Hublet A, De Bacquer D, Valimaa R, Godeau E, Schmid H, Rahav G (2006). Smoking trends among adolescents from 1990 to 2002 in ten european countries and Canada. BMC Public Health.

[5] de Looze M, ter Bogt T, Hublet A, Kuntsche E, Richter M, Zsiros E (2013). Trends in educational differences in adolescent daily smoking across Europe, 2002–10. Eur J Public Health.

[6] Holstein BE, Andersen A, Damsgaard MT, Due P, Bast LS, Rasmussen M. Trends in socioeconomic differences in daily smoking among 15-year-old Danes 1991–2014.Scand J Public Health. 2019. 10.1177/1403494819848284 31074327

[7] Song F, Elwell-Sutton T, Naughton F, Gentry S. Future smoking prevalence by socioeconomic status in England: a computational modelling study.Tob Control. 2020:tobaccocontrol-2019-055490. 10.1136/tobaccocontrol-2019-055490 PMC8237189 32447314

[8] Clare P, Bradford D, Courtney RJ, Martire K, Mattick RP (2014). The relationship between socioeconomic status and ‘hardcore’ smoking over time – greater accumulation of hardened smokers in low-SES than high-SES smokers. Tob Control.

[9] Ministry of Children and Education. Produktionsskoler 2020 [Production schools 2020].

[10] Aarkrog V (2020). The standing and status of vocational education and training in Denmark. J Vocat Educ Train.

[11] Statistics Denmark. Erhvervsudannelser i Danmark 2019 [Vocational schools in Denmark 2019]. Statistics Denmark; 2019.

[12] Jarlstrup NS, Andersen MB, Kjeld SG, Bast LS. §RØG - En undersøgelse af tobak, adfærd og regler: Basisrapport 2020 [§SMOKE – A study of tobacco, behaviour and regulations: Basis report]. 2020.

[13] Aho H, Koivisto A-M, Paavilainen E, Joronen K (2019). The relationship between peer relations, self-rated health and smoking behaviour in secondary vocational schools. Nurs Open.

[14] Perez-Warnisher MT, de Miguel MPC, Seijo LM (2018). Tobacco use worldwide: legislative efforts to curb consumption. Ann Glob Health.

[15] Thomas RE, McLellan J, Perera R (2013). School-based programmes for preventing smoking. Cochrane Database Syst Rev.

[16] Kaftarian S, Robertson E, Compton W, Davis BW, Volkow N (2004). Blending prevention research and practice in schools: critical issues and suggestions. Prev Sci.

[17] Chyderiotis S, Benmarhnia T, Spilka S, Beck F, Andler R, Legleye S (2020). Why do apprentices smoke much more than high school students? Understanding educational disparities in smoking with a Oaxaca-blinder decomposition analysis. BMC Public Health.

[18] Aho H, Koivisto A-M, Paavilainen E, Joronen K (2017). Parental involvement and adolescent smoking in vocational setting in Finland. Health Promot Int.

[19] Lorant V, Rojas VS, Robert P-O, Kinnunen JM, Kuipers MA, Moor I (2017). Social network and inequalities in smoking amongst school-aged adolescents in six european countries. Int J Public Health.

[20] Bast LS, Due P, Bendtsen P, Ringgard L, Wohllebe L, Damsgaard MT (2015). High impact of implementation on school-based smoking prevention: the X: IT study—a cluster-randomized smoking prevention trial. Implement Sci.

[21] Andersen A, Krølner R, Bast LS, Thygesen LC, Due P (2015). Effects of the X:IT smoking intervention: a school-based cluster randomized trial. Int J Epidemiol.

[22] Jøsendal O, Aarø LE, Torsheim T, Rasbash J (2005). Evaluation of the school-based smoking‐prevention program “BE smokeFREE”. Scand J Psychol.

[23] Andersen S, Rod MH, Ersbøll AK, Stock C, Johansen C, Holmberg T (2016). Effects of a settings-based intervention to promote student wellbeing and reduce smoking in vocational schools: a non-randomized controlled study. Soc Sci Med.

[24] Andersen S, Pisinger V, Rod MH, Tolstrup J (2019). Associations of school tobacco policies and legislation with youth smoking: a cross-sectional study of danish vocational high schools. BMJ Open.

[25] Jakobsen GS, Danielsen D, Jensen MP, Vinther JL, Pisinger C, Holmberg T (2021). Reducing smoking in youth by a smoke-free school environment: a stratified cluster randomized controlled trial of Focus, a multicomponent program for alternative high schools. Tob Prev Cessat.

[26] Campbell MK, Piaggio G, Elbourne DR, Altman DG (2012). Consort 2010 statement: extension to cluster randomised trials. BMJ.

[27] Hoffmann TC, Glasziou PP, Boutron I, Milne R, Perera R, Moher D (2014). Better reporting of interventions: template for intervention description and replication (TIDieR) checklist and guide. BMJ.

[28] Bartholomew LK, Parcel GS, Kok G, Gottlieb NH, Fernandez ME (2011). Planning health promotion program: an intervention mapping approach.

[29] Michie S, Hyder N, Walia A, West R (2011). Development of a taxonomy of behaviour change techniques used in individual behavioural support for smoking cessation. Addict Behav.

[30] Ryan RM, Deci EL (2000). Self-determination theory and the facilitation of intrinsic motivation, social development, and well-being. Am Psychol.

[31] Poland BD, Green LW, Rootman I, Poland BD, Green LW, Rootman I (1999). Settings for health promotion: linking theory and practice. Settings for health promotion: linking theory and practice.

[32] Green LW, Kreuter MW (2005). Health program planning: an educational and ecological approach.

[33] The American Heritage Dictionary. Edutainment [Internet]. Date accessed January 6, 2023. Available from: https://www.ahdictionary.com/word/search.html?q=edutainment

[34] Lien N, Friestad C, Klepp KI (2001). Adolescents’ proxy reports of parents’ socioeconomic status: how valid are they?. J Epidemiol Community Health.

[35] Pisinger V, Mikkelsen SS, Bendtsen P, Egan KK, Tolstrup JS. The Danish National Youth Study 2014: Study design, population characteristics and non-response analysis.Scand J Public Health. 2017:1403494817729283. 10.1177/1403494817729283 28914164

[36] Glenstrup S, Bast LS, Danielsen D, Andersen A, Tjørnhøj-Thomsen T (2021). Places to smoke: exploring smoking-related Practices among danish adolescents. Int J Environ Res Public Health.

[37] Danielsen D, Jensen TS, Kjeld SG, Bast LS, Andersen S. Context matters in smoking prevention: evaluating smoke-free school hours in Danish vocational schools. Unpublished manuscript. 10.1093/heapro/daad030 PMC10132565 37099679

[38] Lund L, Lauemøller SG, Kjeld SG, Andersen A, Bast LS (2021). Gender differences in attitudes towards a school-based smoking prevention intervention. Scand J Public Health.

[39] Andersen S, Riis N, Nygart V, Hansen G, Pisinger CH. Rygning på erhvervsskoler-det skal være federe at være ikkeryger [Smoking in vocational schools - it must be cooler to be non-smoker]. Science Council for Prevention; 2018.

[40] The Danish Ministry of Health. Initiativer på tobaksområdet og prisudvikling siden år 2000 [Initiatives on the tobacco area and price development since 2000]. 2020.

[41] Viner RM, Ozer EM, Denny S, Marmot M, Resnick M, Fatusi A (2012). Adolescence and the social determinants of health. Lancet.

[42] Villanti AC, Niaura RS, Abrams DB, Mermelstein R (2019). Preventing smoking progression in young adults: the Concept of Prevescalation. Prev Sci.

[43] Ringgaard L, Heinze C, Andersen N, Hansen G, Hjort A, Klinker C. UNG19-Sundhed og trivsel på erhvervsuddannelser 2019 [Youth 2019 study - Health and well-being at vocational schools 2019].Steno Diabetes Centre Copenhagen, Danish Heart Society, and Danish Cancer Society. 2020.

[44] Helweg-Larsen K, Bøving-Larsen H (2003). Ethical issues in youth surveys: potentials for conducting a national questionnaire study on adolescent schoolchildren’s sexual experiences with adults. Am J Public Health.

