# Supplementary material for: Effectiveness of the multi-component intervention ‘Focus’ on reducing smoking among students in the vocational education setting: a cluster randomized controlled trial
Source: BMC Public Health. 2023 Mar 2;23:419. doi: 10.1186/s12889-023-15331-5 (PMC9979485; doi:10.1186/s12889-023-15331-5)
Supplement: Supplementary file 1 — Supplementary Material 1 [file 12889_2023_15331_MOESM1_ESM.docx]

| **Additional file 1. Description of outcomes** | | | | | | |
| --- | --- | --- | --- | --- | --- | --- |
|  | **Variable name in dataset** | **Question framing** | **Response options** | **Restrictions** | **Recoding** | **Corresponding baseline variable** |
| **Primary outcomes** | | | | | | |
| Number of cigarettes smoked daily | FE8 | How many cigarettes do you approximately smoke during the day? | Range from 1-70 | Smoking status ("Do you smoke cigarettes?") 1 = Yes, everyday |  | BE8 |
| Smoking status: Daily smoking | FE4 | Do you smoke cigarettes? | 1 = Yes, everyday 2 = Yes, at least once a week 3 = Yes, more seldom than every week 4 = No, I do not smoke at present | None | daily_smoking = 1 (FE4=1)  daily smoking = 0 (FE4=2,3,4) | BE4 |
| Smoking status: Regular smoking |  |  |  |  | regular_smoking = 1 (FE4=1,2)  regular smoking = 0 (FE4=3,4) |  |
| **Secondary outcomes** | | | | | | |
| Perceived social benefit effects of smoking (scale) | FE10A | What are your thoughts about smoking…? (Answer as well as you can, even if you do not smoke) – It is easier for students who smoke to develop friendships | 1 = Completely disagree 2 = Disagree 3 = Neither agree nor disagree 4 = Agree 5 = Completely agree | None | Social_benefits = FE10A+FE10E+FE10G Social_benefits_diko = 1 (score above 11) Social_benefits_diko = 0 (score below 11) | BE10A |
|  | FE10E | What are your thoughts about smoking…? (Answer as well as you can, even if you do not smoke) – Students who smoke are more popular |  |  |  | BE10E |
|  | FE10G | What are your thoughts about smoking…? (Answer as well as you can, even if you do not smoke) – Students who smoke look cool |  |  |  |  |
| Perceived mood effects of smoking | FE10D | What are your thoughts about smoking…? (Answer as well as you can, even if you do not smoke)  – Smoking helps when you are bored |  |  | Mood_effects_diko = 1 (FE10D=4,5) Mood_effects_diko = 0 (FE10D=1,2,3) | BE10D |
| Smoking-related self-efficacy | FE9A | Would you be able to not smoke in the following situations…?   - If your friends offer you a cigarette? | 1 = No, I definitely do not think so 2 = No, I do not think so 3 = Yes, I think so 4 = Yes, I definitely think so | Smoking status ("Do you smoke cigarettes?") 1 = Yes, everyday 2 = Yes, at least once a week 3 = Yes, more seldom than every week | Smoking_efficacy = FE9A+FE9B+FE9C Efficacy_diko = 1 (score above 8) Efficacy_diko = 0 (score below 8) | BE9A |
|  | FE9B | Would you be able to not smoke in the following situations…?   - If you are together with friends who smoke? |  |  |  | BE9B |
|  | FE9C | Would you be able to not smoke in the following situations…?   - If you feel nervous? |  |  |  | BE9C |
|  | FE9D | Would you be able to not smoke in the following situations…?  - During the school day? |  |  | FE9D_diko = 1 (FE9D=3,4) FE9D_diko = 0 (FE9D=1,2) |  |
| Intention to initiate smoking | FE15 | Do you think that you will smoke within the next month? | 1 = No, definitely not 2 = No, I do not think so 3 = Yes, I think so 4 = Yes, definitely | Smoking status ("Do you smoke cigarettes?") 4 = No, I do not smoke at present | FE15_diko = 1 (FE15=3,4) FE15_diko = 0 (FE15=1,2) | BE15 |
| Intention to quit smoking | FE11 | Do you want to quit smoking? | 1 = No 2 = Yes, but I have not planned when 3 = Yes, I plan to quit smoking within the next six months 4 = Yes, I plan to quit smoking within the next month | Smoking status ("Do you smoke cigarettes?") 1 = Yes, everyday 2 = Yes, at least once a week 3 = Yes, more seldom than every week | FE11_diko = 1 (FE11=2,3,4) FE11_diko = 0 (FE11=1) | BE11 |
| Nicotine dependence | hsi_fu | HSI was summarized to a score using FE7 (“How soon after you wake up, do you light your first cigarette?”, 0 = more than 60 minutes, 1 = 31-60 minutes, 2 = 6-30 minutes, 3 = Less than 5 minutes) and FE8 ("How many cigarettes do you approximately smoke during a day?" divided into 0 = 1-10 cigarettes, 1 = 11-20 cigarettes, 2 = 21-30 cigarettes, and 3 = more than 31 cigarettes). | Score from 0-6 | Smoking status ("Do you smoke cigarettes?") 1 = Yes, everyday | hsi_fu_cat = 1 (hsi_fu=2,3,4,5,6) hsi_fu_cat = 0 (hsi_fu=0,1,2) | hsi_baseline |
| Smoking during school hours | FE68 | How many cigarettes do you approximately smoke during a day while at school? | Range from 1-30 | Smoking status ("Do you smoke cigarettes?") 1 = Yes, everyday | Numerical variable |  |
| Positive classmate relationships | FE97A | To which extent do you agree/disagree with the following statements…? - Other students accept me as I am | 1 = Completely disagree 2 = Disagree 3 = Neither agree nor disagree 4 = Agree 5 = Completely agree | None | Classmate_relations = FE97A+FE97B+FE97C Classmate_diko = 1 (score above 11) Classmate_diko = 0 (score below 11) |  |
|  | FE97B | To which extent do you agree/disagree with the following statements…? - Most of the students in my class are kind and helpful |  |  |  |  |
|  | FE97C | To which extent do you agree/disagree with the following statements…? - The students in my class enjoy being together |  |  |  |  |
| Sense of class community | FE97D | To which extent do you agree/disagree with the following statements…? - In my class, there is a strong sense of community |  |  | FE97D_diko = 1 (FE97D=4,5) FE97D_diko = 0 (FE97D=1,2,3) |  |
| Perceived student support | FE98A | How often…? - Do you feel that you can get help and support from your classmates, when you need it | 1 = Very often 2 = Often 3 = Sometimes 4 = Almost never 5 = Never | None | FE98A_diko = 1 (FE98A=1,2) FE98A_diko = 0 (FE98A=3,4,5) |  |
| Perceived teacher support | FE98B | How often…? - Do you feel that you can get help and support from your teachers, when you need it |  |  | FE98B_diko = 1 (FE98B=1,2) FE98B_diko = 0 (FE98B=3,4,5) |  |
| Afraid of being made a fool of | FE99 | In your class, are you afraid of being made a fool of? | 1 = Yes, very often 2 = Yes, often 3 = Yes, sometimes 4 = No | None | FE99_diko = 1 (FE99=1,2) FE99_diko = 0 (FE99=3,4,5) |  |
| School connectedness | FE100 | Think of your school in general. To which extent do you agree/disagree with the following statement: ‘I feel that I belong at my school’? | 1 = Completely disagree 2 = Disagree 3 = Neither agree nor disagree 4 = Agree 5 = Completely agree | None | FE100_diko = 1 (FE100=4,5) FE100_diko = 0 (FE100=1,2,3) |  |
| Socializing around smoking | FE67A | How often do you smoke during the school day (with others or alone)…? - With teachers at school | 1 = Everyday 2 = Several days a week 3 = More seldom 4 = Never | Smoking status ("Do you smoke cigarettes?") 1 = Yes, everyday 2 = Yes, at least once a week 3 = Yes, more seldom than every week | FE67A_diko = 1 (FE67A=1,2) FE67A_diko = 0 (FE67A=3,4) |  |
|  | FE67B | How often do you smoke during the school day (with others or alone)…? - With other students at school |  |  | FE67B_diko = 1 (FE67B=1,2) FE67B_diko = 0 (FE67B=3,4) |  |
|  | FE67C | How often do you smoke during the school day (with others or alone)…? - Alone at school |  |  | FE67C_diko = 1 (FE67C=1,2) FE67C_diko = 0 (FE67C=3,4) |  |
| Less visibility of smoking | FE20C | How many at the school do you see smoke daily...? - Among all the students at school | 1 = None 2 = Few/a couple 3 = Less than half 4 = Half 5 = Most 6 = All | None | FE20C_diko = 1 (FE20C=4,5,6) FE20C_diko = 0 (FE20C=1,2,3) |  |
|  | FE20D | How many at the school do you see smoke daily...? - Among your classmates |  |  | FE20D_diko = 1 (FE20D=4,5,6) FE20D_diko = 0 (FE20D=1,2,3) |  |
| Knowledge about smoking cessation support | FE12 | Do you know any smoking cessation options that could help you smoke less (e.g., course, app, or phone counseling)? | 1 = Yes, I know one or more options I would like to use 2 = Yes, I know one or more options, but I do not want to use them 3 = No | Smoking status ("Do you smoke cigarettes?") 1 = Yes, everyday 2 = Yes, at least once a week 3 = Yes, more seldom than every week | FE12_diko = 1 (FE12=1) FE12_diko = 0 (FE12=1,2) | BE12 |
| Denormalized (descriptive) social norms for smoking at school (and class) level | FE77 | How many students at your school do you think smoke daily? | 1= None 2 = 10 % 3 = 20 % 4 = 30 % 5 = 40 % 6 = 50 % 7 = 60 % 8 = 70 % 9 = 80 % 10 = 90 % 11 = All | None | FE77_diko = 1 (FE77=7,8,9,10,11) FE77_diko = 0 (FE77=1,2,3,4,5,6) |  |
| **Covariates and subgroup analyses** | | | | | | |
| Age | age | Age – estimated by birth date | Age in integers | None | Numerical variable |  |
| Smoking status | smokingstat_  baseline | Smoking status at baseline. Categorized based on two variables: BE4 (Do you smoke cigarettes?) and BE14 (Have you previously smoked?) | 1 = Daily smoking 2 = Occassional smoking  3= Previously smoking 4 = Tried smoking once or twice 5 = Never smoked | None | smokingstat_baseline = 1 (BE4=1) smokingstat_baseline = 2 (BE4=2,3) smokingstat_baseline = 3 (BE4=4 and BE14=1,2,3) smokingstat_baseline = 4 (BE4=4 and BE14=4) smokingstat_baseline = 5 (BE14=4 and BE14=5) |  |
| Gender | gender | Are you male or female? | 1 = Male 2 = Female | None |  |  |
| Socioeconomic status | osc | Variable assessing Family Occupational Social Class (OSC). Recoding of the social group coding (SES) to four categories. SES | 1 = High 2 = Middle 3 = Low  4 = Unclassifiable | None | OSC = 1 (SES=1,2) OSC = 2 (SES=3,4) OSC = 3 (SES=5,7) OSC = 4 (SES=6,8) |  |
| School type | schooltype | Three school types (CHP schools, other VET schools, PBE) | 1 = CHP 2 = Other VET schools 3 = PBE | None |  |  |
