# Supplementary material for: Effectiveness of the multi-component intervention ‘Focus’ on reducing smoking among students in the vocational education setting: a cluster randomized controlled trial
Source: BMC Public Health. 2023 Mar 2;23:419. doi: 10.1186/s12889-023-15331-5 (PMC9979485; doi:10.1186/s12889-023-15331-5)
Supplement: Supplementary file 2 — Supplementary Material 2 [file 12889_2023_15331_MOESM2_ESM.docx]

| **Additional file 2. Sensitivity analysis: General linear regression analyses of the associations between the intervention (versus control group) and daily cigarette consumption at follow-up using multiple imputation of missing data, included if daily number of cigarettes are <25 (40xN=494-470^a^).** | | | | | | | |
| --- | --- | --- | --- | --- | --- | --- | --- |
| **Outcomes** | **Adjusted*** | | | **Fully adjusted**** | | |  |
| **Daily number of cigarettes** | **Mean difference^b^** | **CI (95%)** | **p-value** | **Mean difference^b^** | **CI (95%)** | **p-value** |  |
| Control group | 0 |  |  | 0 |  |  |  |
| Intervention group | -0.12 | -1.02;0.77 | 0.78 | -0.14 | -1.04;0.76 | 0.76 |  |
| *adjusted for baseline smoking status/consumption | | | | | | | |
| **adjusted for baseline smoking status/consumption, gender, socioeconomic status, and age  ^a^number differed due to the multiple imputation models | | | | | | | |
| ^b^mean difference in change from baseline to follow-up | | | | | | | |
